# Herpetic Keratouveitis and Trabeculectomy Failure during Infliximab Therapy in a Patient with Behçet’s Disease

**DOI:** 10.4274/tjo.59354

**Published:** 2016-04-05

**Authors:** Sirel Gür Güngör, Leyla Asena, Ahmet Akman, Onur Gökmen

**Affiliations:** 1 Başkent University Faculty of Medicine, Department of Ophthalmology, Ankara, Turkey

**Keywords:** Behçet’s disease, herpetic keratouveitis, infliximab, trabeculectomy

## Abstract

A 51-year-old man was diagnosed with Behçet’s disease in 2001. The patient was resistant to all immunosuppressive therapies. After 6 months of infliximab therapy, he presented to our clinic with pain and blurred vision in his right eye. The visual acuity was 20/200 and the intraocular pressure was 35 mmHg in the right eye. Biomicroscopic examination revealed corneal dendritic ulcers and 2+ cells in the anterior chamber in the right eye. The herpetic keratouveitis attack was controlled with antiviral therapy but the patient needed another glaucoma surgery. Trabeculectomy with mitomycin C was performed about halfway through an eight-week interval between two doses of infliximab.

## INTRODUCTION

Behçet’s disease (BD) is a serious sight-threatening clinical entity that involves uveitis accompanied by recurrent oral aphthous ulcers, genital ulcers, skin lesions, and other systemic lesions. Although infliximab, a humanized antibody against tumor necrosis factor-alpha (TNF-α), reduces uveitis attacks in patients with BD, anti-TNF-α therapy increases the risk of infections due to the systemic blockade of TNF-α.^1,2,3^

Here, we report herpetic keratouveitis triggered by treatment with the anti-TNF-α antibody infliximab in a uveitis patient with BD and the failure of the previous trabeculectomy during the course of the infection. To our knowledge, this is the first reported case of new-onset herpetic keratouveitis triggered by anti-TNF-α therapy in a patient with BD.

## CASE REPORT

A 51-year-old man was diagnosed with BD in 2001. Despite treatment with 5 mg/kg cyclosporine, 3 mg/kg azathioprine and corticosteroids, the patient experienced frequent and severe panuveitis attacks in both eyes. The patient was resistant to all treatments including interferon alpha and mycophenolate mofetil. The patient underwent phacoemulsification surgery in the right eye in 2004 and trabeculectomy in the same eye in 2006. Almost all systemic side effects were a result of steroid usage.

**Figure 1:**
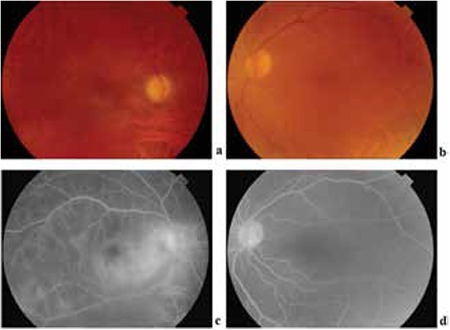
In the posterior segment examination, vitreous haze was observed in both eyes (a, b). Fundus fluorescein angiography revealed vascular leakage and cystoid macular edema in the right eye (c). There was no vascular leakage in the left eye (d)

The patient suffered a bilateral panuveitis attack in January 2012 (Figure 1). The visual acuity decreased to 20/400 in the right eye and 20/40 in the left eye. He was started on infliximab in February 2012. Infliximab therapy at 5 mg/kg was administered at 0, 2, and 6 weeks, and every 8 weeks thereafter. Because he experienced side effects related to azathioprine and cyclosporine, the patient reduced the dose of the drugs and subsequently self-terminated the therapy. The visual acuity was 20/40 in the right eye and 20/25 in the left eye in the third month of infliximab therapy. The intraocular pressure was 14 mmHg in both eyes. The anterior and posterior segments were quiet in both eyes.

**Figure 2:**
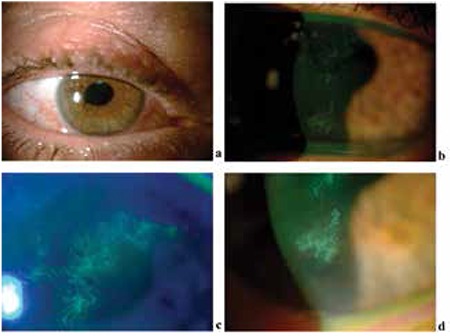
Biomicroscopic examination revealed mild ciliary injection (a) and corneal dendritic ulcers in the right eye (b, c, d)

**Figure 3:**
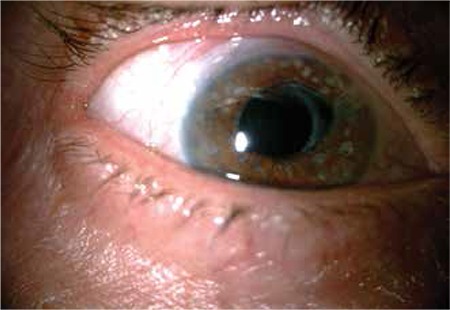
Six months after the keratouveitis attack, biomicroscopic examination revealed a clear cornea, patchy iris defects and two peripheral iridectomies

After 6 months of infliximab therapy, he presented to our clinic with pain and blurred vision in his right eye. The visual acuity was 20/200 in the right eye and 20/25 in the left eye. The intraocular pressure was 35 mmHg in the right eye and 16 mmHg in the left eye. Slit-lamp examination revealed corneal dendritic ulcers and 2+ cells in the anterior chamber in the right eye (Figure 2). There was no vitritis or vitreal flare. The posterior segment was quiet. There was no inflammation in the left eye. This was the first herpetic keratitis or keratouveitis attack the patient had experienced. Treatment with 800 mg oral acyclovir twice daily, topical acyclovir pomade five times daily, and topical brimonidine combined with dorzolamide/timolol fixed combination was started. After 5 days, the corneal ulcers had regressed but the anterior chamber inflammation persisted. Therefore, topical prednisolone five times daily was added to the therapy. His intraocular pressure was still 31 mmHg. After 2 weeks the corneal ulcers healed, patchy iris atrophies were detected and the anterior chamber reaction was under 1+ (Figure 3), so the topical acyclovir therapy was discontinued. Topical prednisolone acetate was gradually tapered as the anterior chamber inflammation disappeared. His intraocular pressure was still over 30 mmHg and a repeat trabeculectomy with mitomycin C was planned. In the first month after the herpetic keratouveitis attack, trabeculectomy with mitomycin C was performed about halfway through an eight-week interval between two doses of infliximab. Neither ocular inflammatory attacks nor infectious complications were observed in the operated eye during postoperative follow-up with the use of acyclovir 800 mg/day. We attempted to gradually reduce the acyclovir dose but anterior chamber reaction was observed at doses lower than 800 mg.

Approximately 10 months after the operation, the patient discontinued the acyclovir therapy and he presented again to our clinic with ocular discomfort. A small corneal dendritic ulcer and 1+ cell reaction in the anterior chamber were observed on slit-lamp examination. Systemic acyclovir 800 mg/day and acyclovir pomade 5 times a day were reinitiated. The corneal ulcers and the anterior chamber inflammation were suppressed rapidly within a few days.

The patient has not experienced any further uveitis attacks due to herpes virus or BD during follow-up; the patient’s right eye is currently in a good condition without inflammation in the anterior chamber for the last four months. His present right visual acuity is 20/30 and the intraocular pressure is around 12 mmHg. He is still receiving infliximab therapy and prophylactic oral acyclovir at 800 mg/day.

## DISCUSSION

Infliximab is a humanized antibody against TNF-α that can greatly reduce ocular inflammatory attacks in uveitic patients affected by BD. However, anti-TNF-α therapy is also associated with a risk of infectious complications due to the systemic blockade of TNF-α. In addition, patients with BD may frequently need ocular surgery. Therefore, intraocular surgery in BD patients under treatment with infliximab may be associated with a higher risk of ocular infections.^4^ Moreover, it is unclear if trauma caused by surgery increases disease activity during anti-TNF-α treatment. Trabeculectomy with mitomycin C has provided long-term safety and was effective in reducing intraocular pressure in cases with secondary glaucoma associated with BD.^5,6^ Trabeculectomy has also been successful in patients with BD receiving infliximab therapy.^7^

In addition to bacterial and fungal infections, biologic treatments may also increase the risk of viral infections; however, previous studies regarding this issue have not been conclusive enough.8 There are some case reports and studies which reported that herpes zoster infections developed in patients with rheumatologic disease during the biologic treatment course.^8,9,10,11,12,13^ There are also some reported cases of cutaneous herpes simplex virus (HSV) infection following treatment with infliximab.^14,15^ We are not aware of any published reports of ocular HSV infections associated with use of TNF inhibitors.

In vivo data indicate that TNF-α may have an antiviral effect in HSV-1 infections. In a model in which HSV-1 was reactivated in latently infected mouse cornea, TNF-α and interleukin-6 were the predominant cytokines within the trigeminal ganglion, suggesting a key role for these cytokines in viral clearance.^16^ Absence of TNF in knockout mice increased susceptibility to primary corneal HSV-1 infections in one study^17^ and lowered survival rates compared with wild-type mice in another.^18^ While all three TNF inhibitors used in clinical practice inhibit the actions of TNF-α, their different mechanisms of action may result in a variable susceptibility to HSV-1 infections, although this has not specifically been studied.

In several small placebo-controlled trials, prophylactic use of oral acyclovir in immunocompromised patients was found to be successful in reducing the duration of viral shedding and preventing clinical HSV infections in 80% to 100% of patients.^19^ The oral doses studied were 600 mg/day for 30 days and 800 mg/day for 180 days. In both studies, there were no additional adverse events compared with placebo. The most frequently reported adverse effects during acyclovir therapy are headache, nausea and abdominal cramping. Although oral acyclovir has a good safety profile, cases of rapidly progressive acute neurological and renal toxicity have been described.^20^ Acyclovir-induced neurotoxicity can present with a variety of symptoms including agitation, delirium and hallucinations.^21^ Dose reductions are recommended in patients with renal impairment and in the elderly. Our patient received acyclovir at 800 mg/day for 24 months and a dose reduction was attempted, inflammation in the anterior chamber developed. Although current evidence concerning the optimum duration and dose for long-term prophylaxis is lacking, a decision to continue at this level of therapy was made with the patient because of concerns about infection recurrence.

In our patient, herpetic keratouveitis occurred under infliximab therapy, and the previous trabeculectomy surgery failed due to this attack. The keratouveitis attack was controlled with antiviral therapy but the patient needed repeated glaucoma surgery. The trabeculectomy surgery was risky in this patient because the surgery might have induced both BD and herpetic uveitis; in addition to that, infection was another risk. After the glaucoma surgery under systemic antiviral and infliximab therapy, there were no occurrences of inflammatory or infectious complications.

## CONCLUSION

In conclusion, systemic and ocular infections including HSV infections and reactivations can develop in patients receiving immunosuppressive or biologic agents; therefore, these patients should be monitored closely.

## Ethics

Informed Consent: Received.

Peer-review: Externally peer-reviewed.
